# Parental Bonding and Children’s Psychopathology: A Transgenerational View Point

**DOI:** 10.3390/children8111012

**Published:** 2021-11-05

**Authors:** Alessia Raffagnato, Caterina Angelico, Rachele Fasolato, Eleonora Sale, Michela Gatta, Marina Miscioscia

**Affiliations:** 1 Child and Adolescent Neuropsychiatric Unit, Department of Women’s and Children’s Health, University Hospital of Padua, 35128 Padua, Italy; caterina.angelico@gmail.com (C.A.); rachele.fasolato@gmail.com (R.F.); michela.gatta@unipd.it (M.G.); marina.miscioscia@unipd.it (M.M.); 2 Child and Adolescent Neuropsychiatric Unit, ULSS6 Padua, 35143 Padua, Italy; Eleonora.sale@aulss6.veneto.it; 3 Department of Developmental Psychology and Socialization, University of Padua, 35131 Padua, Italy

**Keywords:** parental bonding, transgenerational view, adolescent’s psychopathology, developmental psychopathology

## Abstract

Literature confirmed parental bonding as one of key factors influencing offspring’s psychopathology; the present study aimed to investigate, with a case-control study, the relationship between parental bonding and psychopathology in an Italian adolescent sample. The clinical sample was composed of 64 adolescents from 12 to 18 years old (M_age_ 15.00; S.D. 1.70) attending a Neuropsychiatric Unit of Veneto; the non-clinical sample was composed of 61 adolescents, from 13 to 18 years old (M_age_ 14.80; S.D. 1.32) attending middle and high school in the province of Padua and Pesaro (Italy); their parents (mothers and fathers) were also involved. In the study, self-reported tests were administered (Parental Bonding Instrument, Child Behavior Checklist, Youth Self Report). Our study confirmed a correlation between parental bonding and adolescent psychopathology: dysfunctional parenting styles (characterized by low care and high control) were more frequent among cases in contrast to controls. An effect of gender also appeared. In the Italian adolescent clinical sample, parental bonding, especially low parental care, was correlated to the emergence of psychopathology.

## 1. Introduction

Mental health problems affect 10–20% of children and adolescents worldwide [1]. A wider Italian prevalence study used the PrISMA study, and found that 9.8% (Confidence Interval (CI) 8.8–10.8%) (Child Behavior Checklist caseness) or the 8.2% (CI 4.2–12.8%) (Diagnostic and Statistical Manual of mental disorders-fourth edition disorders) of Italian pre-adolescents met psychopathologist criterions [2].

Several studies investigated the causes of mental illness, but until now, risk and protective factors are the actual models investigated. Parenting function is assigned high relevance among these factors, and it can assume both a positive role, increasing the child’s resilience, or a negative role representing a risk factor that contributes to the vulnerability of the individual.

Many studies examined the influence of genetic factors on the development of psychopathology [3], studying, for instance, intergenerational transmission of risk for depression [4], the influence of conduct disturbance [5], or the development of high-risk behaviors, including self-injury [6]. Many factors contribute to an individual’s vulnerability, both genetic and environmental influences explain the association between parental function and offspring well-being [7]. Since there is a strong relationship between genetic and environmental factors, some authors hypothesized that symptoms in adolescence may influence parental symptoms due to the evocative gene–environment correlations (evocative rGE), which happens when adolescents’ genetically-influenced traits or symptoms elicit environmental responses from others [8,9].

Between the several functions that determine parenting style, Parker [10] identified two specific characteristics of parenting: care and overprotection. These can be used to describe four parental bondings: optimal (high care and low overprotection); weak/absent (low care and low overprotection); affectionate constraint (high care and high overprotection); affectionless control (low care and high overprotection) [10].

In particular, the dimension of parental care contrasts with indifference and rejection, while the dimension of overcontrol hinders with the ability to be independent [10].

Literature found a strong correlation between parental bonding and psychopathological disorders in adults, such as depression [11,12], anxiety disorders [13,14], obsessive-compulsive disorders [15], and schizophrenia [16,17], which were more frequently associated with the subscale affectionless control bonding. Patients affected by hypochondriasis or panic disorders reported an affectionate constraint bonding [15]. The correlation between the “optimal” bonding and lower level of stress and higher social support and wellness was also demonstrated [18]. Furthermore, it has been demonstrated that low care represents a great risk factor for the emergence of psychopathology [19,20]; studies demonstrated that dysfunctional parental bonding is more frequent in psychopathology-affected populations, both in adults [13,19,20,21,22] and adolescents [23,24].

Bolbwy’s studies on attachment theory [25] provide an important contribution in understanding the link between attachment and psychopathological vulnerability, and to comprehend intergenerational transmission. Many studies in reference to Bowlby’s theoretical framework, investigated the relationship between attachment styles and psychopathological symptoms. Lacasa and colleagues [26], in a case-control study, found a similar relationship for clinical and non-clinical adolescents, specifically highlighting a link between preoccupied attachment style, internalizing and externalizing symptoms, somatic complaints, anxious–painful behavior, verbal aggression, attention-seeking behavior, and thinking problems.

As for affective syndromes, child anxiety disorder has been associated with parental overcontrol [27] and parental perfectionism [28], low parental warmth correlated with depression in adolescent [29]. The perception of the quality of maternal care received during infancy was negatively associated with emotional eating in a group of adolescents and young adults [30], while mother–child relationship quality was negatively associated with loneliness and depressive symptoms in adolescence [31].

McKinney and colleagues [32] showed that adolescent irritability correlated negatively with parental warmth and positively with parental overprotection.

The dimension of low parental care and high overprotection was also significantly correlated with individuals at high risk for psychosis [33] where parental overprotection and control discouraged the achievement of independence and autonomy [34].

Eun and colleagues [35] examined the association between parenting styles and adolescent mental disorders, highlighting gender differences. The authors showed that high maternal care and low maternal control was a protective factor for depressive, eating and behavioral disorder, while high paternal care and low paternal control was a protective factor for social phobia and alcohol abuse or dependence. The authors found gender differences in association; for example, high maternal control was correlated, only for females, with anxiety and substance-use disorders, while in only males, high paternal control correlated with substance-use disorders.

Beyond sex difference, “controlling–indulgent parenting” was associated with greater odds of adverse child outcomes [36].

Abbaspour and colleagues [37] investigated parenting styles of patients with schizophrenia, depression, and bipolar disorder. The study found high levels of control in patients with bipolar disorder compared with other disorders. The majority of patients described dysfunctional paternal parenting styles, with predominantly low levels of care and protection (neglectful parenting). Regarding maternal parenting style, significant differences between disorders were found. In patients affected by schizophrenia, the ineffective style of mothers most frequently found was affectionate constraint (high care and protection). In mood disorders, low levels of maternal care and protection prevailed.

A longitudinal study by Hou and colleagues [3] found that parents’ depressive symptoms, negative parenting strategies (low nurturant-involved parenting) and depressive symptoms of an adolescent child influenced each other in a vicious circle. In particular, maternal depressive symptoms were significant predictors of adolescent depressive symptoms, as opposed to paternal depressive symptoms; this was due in part to the different parenting role in the development of their adolescent child. However, depressive symptoms in adolescents seemed to influence and worsen child–parent interaction, which in turn could cause depressive symptoms in parents themselves; the mother–child relationship is even more difficult as maternal depression reinforced depressive symptoms in the adolescent child [3].

Li and colleagues [38] investigated the intergenerational transmission of grandparents’ parenting on children’s internalizing problem and showed a significative association between them. They found that psychological control perceived by parents influenced children emotional regulation.

Grandparental care and overprotection were associated with the child’s emotional and behavioral problems, mediated by parenting styles [39]. Jiménez-Iglesias and colleagues [40] showed that dimensions of promotion of autonomy, shared activities with the family, and above all parental affection, were protective factors for health-related quality of life in adolescents.

Dysfunctional parental bonding could increase the risk of emotional dysregulation that appears in adulthood as insecure attachment and alexithymia [41] and could be a risk factor for the need for independence and socialization [38] that assume great importance among the developmental tasks in adolescence. Particularly, alexithymia has been found to be related to parent–child relationships and is defined as a vulnerability factor for different somatic, emotional, and behavioral problems [42,43,44]. It is then important, both in physiological and clinical environments, to use efficacy tools aimed to evaluate the child within the family context [45,46].

The child–parent relationship has also been investigated in neuroimaging studies; in particular, Van der Cruijsen and colleagues [47] investigated neural indicators of mother–adolescent relationships in a group of adolescents, and found a particular activation of medial prefrontal cortex in relation to close mother–adolescent relationships with emotional closeness [3,47], in line with previous fMRI studies that showed similar activation in the medial prefrontal cortex in the evaluation of self and near-others, indicative of relations of closeness.

### Aims of the Study

The study was part of a broader project about family interactions and it was approved by Ethical Commission of Padua Hospital (CESC, 6.4.17). In this case-control study, for the first analysis, we wanted to investigate the relationship between parental bonding and psychopathology in an Italian adolescent sample and observe the influence of offspring gender. In particular, we also wanted to investigate the influence of parental care and parental control on internalizing and externalizing problems.

Thus, our second objective was to verify the assumption that clinical and non-clinical populations differed in terms of parent–child relationships, both in the present and in a trans-generational perspective (i.e., the bond established with their child compared to one they themselves experienced as children).

In particular, comparing parental relationships detected by the children’s Parental Bonding Instrument (PBI) with those detected by parents’ PBIs, we expected that cases would differ from controls, and that the child’s gender would influence these results. Although some studies on investigations of generational transmission of parental bonding have considered both maternal and paternal figures [48,49,50], to the authors knowledge there is no literature on how the two parental relationships relate to each other; therefore, we wanted to assess how the degree of care/control perceived by the child by one of the parents varied according to that of the other parent, expecting that this would differ in the two groups (cases and controls) and that this would also occur in the parental couples.

## 2. Materials and Methods

### 2.1. Sample

We enrolled a case sample of 64 adolescents (26 males and 38 females, from 12 to 18 years old, M_age_ = 15.00, SD = 1.70) accessing the neuropsychiatric outpatients’ services for adolescents from January 2018 to June 2019 for a psychodiagnostic consultation. We also enrolled a control sample of 61 adolescents (32 males and 29 females, from 13 to 18 years old, M_age_ = 14.80, SD = 1.32) attending 2 middle and 2 high schools in the province of Padua. Participation required informed consent of the parents and the adolescents. The inclusion criteria for study participants were as follows: adolescent age (more or equal than 11 years old and lower than or equal to 18 years old) and obtaining informed consent of the parents. Moreover, inclusion criteria for the case group also included at least one access to the neuropsychiatric outpatients’ services. Socio-economic status (SES) was assessed through the Hollingshead index (13) for the case sample and results were normally distributed; in the control sample, SES was assumed distributed as in the general population. For reason of simplicity and in accordance with scientific nomenclature, adolescents were referred to as the G2 generation, and the parents as the G1 generation.

### 2.2. Materials

#### 2.2.1. Parental Bonding Instrument (PBI)

Using the Parental Bonding Instrument, adolescents were asked to recall how they were parented by their mother and father “when they were children”. The 25 items, assessing the domains of care (12 items) and overprotection (13 items), were scored on a 4-point Likert type scale, ranging from 0 = “very unlike” to 3 = “very like.” For some items, the scale was reversed scored based on the wording of the item. An example of an item scored on the “care” domain is “could make me feel better when I was upset;” an item scored on the “overprotection” domain is “tried to control everything I did.” Participants were required to respond to items for mothers and fathers separately. Scores for each domain represented the sum total of domain items and could range from 0–36 on the parental care, and 0–39 in terms of parental overprotection. According to cutoff points established by Scinto [51], scores below 28/25 indicated low maternal/paternal care, while scores above 15/13 indicated high maternal/paternal overprotection. The PBI was evaluated extensively for its psychometric properties, and has been used with a variety of populations and has demonstrated good retest reliability, internal consistency, and validity [15]. PBI has been used for adolescents in other studies [52].

#### 2.2.2. Youth Self Report (YSR) and Child Behavior Checklist (CBCL)

The present study used the latest YSR 11–18 and CBCL 6–18 version [53]. Items were scored from 0 to 2 (0 = not true, 1 = somewhat or sometimes true, 2 = very true or often true) on the basis of the preceding 6 months. Problems items could be scored on narrow-band scales: Withdrawn, Somatic Complaints, Anxious/Depressed, Social Problems, Attention Problems, Thought Problems, Delinquent Behavior and Aggressive Behavior; or on broad-band scales: Internalizing and Externalizing Problems, and the Total Problems scale. T-scores were considered, and on the basis of the Achenbach System of Empirically Based Assessment (ASEBA) cut off, we determined 3 groups: non-clinical, borderline, and clinical. Borderline and clinical t-scores made up a unique group (named Clinical).

### 2.3. Statistical Analyses

Clinical comparisons were performed using t and Chi-square tests; care/overprotection within the parental couple were investigated using correlation. Statistical analyses were done using SPSS software from IBM (IBM SPSS Statistics for Windows, Version 24.0. Armonk, NY, USA). All tests were two-sided with significance set at 0.05.

## 3. Results

### 3.1. Parental Bonding’s Distribution

First, we evaluated the distribution of parental bonding in cases and in controls, applying a chi-square test. As shown in Table 1, paternal bonding was non-randomly distributed (χ^2^(3) = 15.174; *p* < 0.01). More in detail, the majority of cases (34.55%) reported “affectionless control” whereas almost half of controls (47.54%) reported an “optimal” bonding with their fathers. Maternal bonding were non-randomly distributed (χ^2^(3) = 8.897; *p* < 0.05). The majority of cases (36.36%) reported a “weak/absent” bonding; “optimal” bonding was the most frequently reported by controls (42.62%). The cases reported bonding, both maternal and paternal, characterized by low care.

We executed a bidirectional homoscedastic *t*-test, for independent samples, to compare the average score of PBI of the two groups. The results are presented in Table 2. For both paternal and maternal “care dimension”, the average scores resulted as statistically different between cases and controls (*p* < 0.01); regarding “overprotection dimension”, the difference was not statistically significant.

### 3.2. Gender Influence

To evaluate the gender influence, we applied a *t*-test for independent samples, between female and male subgroups of cases and controls. As shown in Table 3, for the female gender, cases and controls differed by the care dimension, both paternal and maternal (*p* < 0.01). Moreover, they differed in terms of maternal overprotection (*p* = 0.05). Girls of case samples perceived a lower care (maternal and paternal) and a higher maternal overprotection. In the male gender, there was no statistical difference between cases and controls, nor was there for the care dimension.

### 3.3. Association between PBI and Psychopathology

Regarding YSR scores, it was possible to distinguish four groups: individuals with internalizing problems (anxiety, depression, somatic symptoms), individuals with externalizing problems, individuals with total problems, and individuals with normal t-scores. We analyzed the distribution of the parental bonding among the four groups, applying a chi-square test. The results are presented in Table 4. Paternal bonding were not randomly distributed (χ^2^ (9) = 21.513, *p* < 0.05): “Absent/weak” bonding was the most reported by the individuals with externalizing problems (46.15%); 52.94% of individuals with total problems reported “affectionless control”, moreover high overprotection was never associated to high care (0% affectionate constraint); individuals with normal T-scores (YSR-score), reported more often “optimal” bonding (47.69%). Maternal bonding were not randomly distributed (χ^2^ (9) = 21.337, *p* < 0.05): 42.11% of individuals with internalizing problems reported “weak/absent” bonding and 31.58% an “optimal” bonding, both characterized by a low overprotection; 46.15% of individuals with externalizing problems reported “weak/absent” bonding; that was also the most reported by individual with total problems (58.83%); (47.69%) non-pathologic individuals reported “optimal” bonding.

We grouped the participants on the basis of CBCL score, identifying four groups (individuals with normal t-scores, internalizing problems, externalizing problems, total problems), and applying a Chi-square test we evaluated the distribution of parental (both paternal and maternal) bonding defined through the PBI. Concerning the paternal and maternal bonding, the test result was not significant (χ^2^ (9) = 10.886; χ^2^ (9) = 11.762).

### 3.4. Parental Bondings of Parents

We compared parents of cases and parents of controls (G1) to assess difference in parental bonding between them about control and care. A two-tailed, independent-samples Student’s *t*-test was then performed between the mean scores that parents reported by PBI on their relationship with their parents. As shown in Table 5, the difference in mean PBI scores between case and control fathers did not differ significantly from each other. Analyses regarding mothers showed that the mean PBI score related to maternal care was statistically significantly higher in mothers of controls than in mothers of cases.

### 3.5. Transgenerational Trasmission of Parental Bonding

We compared parental bonding of children (G2) and parents (G1) to assess whether the type of bonding persisted from one generation to the next in the whole sample. A two-tailed Student’s *t*-test for independent samples was then applied to the averages of the child and parent PBI scores. The results are presented in Table 6.

### 3.6. Transgenerational Trasmission of Parental Bonding: Influence of Gender

Controls differed from parents in terms of care, whereas cases differed from parents in terms of control. We therefore assessed whether the relationship was equally valid in the female and male subgroups by means of a two-tailed Student’s *t*-test for independent samples, which compared the mean PBI scores of G2 generation with those of G1 generation. The results are shown in Table 7.

### 3.7. Matching the Degree of Care/Control between Mother and Father

In order to compare the degree of care/control between mothers and fathers, a Pearson correlation test was carried out between the scores for fathers and mothers on the PBI completed by children (G2) and parents (G1), observing, in particular, what happened in the transgenerational transition.

Controls: In controls group, there was a positive linear correlation between mothers’ and fathers’ scores, related to care, both when PBI was filled out by the child (*ρ* = 0.661; *p*-value = 0.001 **) and by the parents (fathers *ρ* = 0.490; *p*-value = 0.001 **; mothers: *ρ* = 0.507; *p*-value = 0.001 **). A similar result was obtained for the variable “control” when PBI was carried out by the child (*ρ* = 0.658; *p*-value = 0.001 **) and by the parents (fathers *ρ* = 0.519; *p*-value = 0.001 **; mothers *ρ* = 0.447; *p*-value = 0.001 **).

Cases: Children did not report a correlation between maternal and paternal care (*ρ* = 0.185; *p*-value = 0.172), unlike parents who instead showed a linear correlation (fathers *ρ* = 0.657; *p*-value = 0.001 **; mothers *ρ* = 0.432; *p*-value = 0.001 **). Maternal and paternal control correlated when reported by the children (*ρ* = 0.445; *p*-value = 0.001 **) and mothers (*ρ* = 0.646; *p*-value = 0.001 **), while they did not correlate when reported by fathers (*ρ* = 0.183; *p*-value = 0.208).

## 4. Discussion

The present case-control study aimed to investigate the relationship between parental bonding and psychopathology in an Italian adolescent sample and observe the influence of offspring gender. The results showed differences between cases and controls in the distribution of parental bonding. Specifically, controls showed a higher percentage/rate of optimal maternal and paternal bonding while cases reported more paternal affection control and maternal absent bonding. The results showed that the care dimension was what made the difference: adolescents in the case group perceived less care from both parents. Low care is a great risk factor for the emergence of psychopathology [3,19,20,29,30], while optimal bonding is more associated with the absence of psychopathology [13,14,18,19,21,23,24].

Adolescence is a period of change when it is important become independent of parents, so overprotection should be modified and reduced to allow the child to develop self-confidence; nonetheless, care still seems to have a crucial role [34]. Obviously, during adolescence, care cannot be only through material actions (provide food, home, personal hygiene) which are essential during childhood, but the parent must give a different kind of care, more mature, assuming the role of affection–giver and so the child can find support of their values and capacities.

The literature highlighted how, with regard to depressive symptoms, parental psychopathological symptoms, negative parenting skills, and depressive symptoms in the children themselves were linked in a sort of vicious circle, thus emphasizing the importance of parental training to reduce negative parenting, especially in families at higher risk of depression [3], in which both genetic and environmental factors contribute to the development of psychopathology [4,5,7]. The literature demonstrates that “affectionless control” was more frequent in drug-addicted patients [54,55] and in schizophrenics [16,17]. In an Israeli adolescent sample, “affectionless control” was associated to psychopathological problems or to lesser wellbeing [18].

Affectionless-controlling or affectionless-authoritative-overprotective styles on PBI may be associated with a high risk of psychosis [33].

Considering only the dimensions of parenting, valuated through CRPBI (Child’s Report of Parental Behavior [18,19]) is a self-reported questionnaire of 56 items, it identifies not two, but three dimensions of parental bonding: care, behavioral control, psychological control), and not the parental bonding, Galambos and colleagues demonstrated that externalizing problems in an adolescent population was correlated to high psychological control [23]; Bean and colleagues demonstrated that paternal care were always inversely correlated to depression in young African-Americans [24].

While ineffective paternal parenting with low levels of care and protection were found in most patients with mood disorders (depressive and bipolar) and schizophrenia, low levels of maternal protection and care prevailed in mood disorders, ineffective maternal parenting related to affectionate constraint in schizophrenia and high levels of parental control were found in patients with bipolar disorder [37].

In previously cited studies that affirmed the relationship between parenting and psychopathology, data were not examined on the basis of child gender, and samples were basically homogenous but with a slight female prevalence (from 54 to 61%) [10,18,23,24] and only in a few cases was there a male prevalence [37,56,57]. Other studies examined the gender influence. The relationship between parenting, discipline and emotional adjustment is different between boys and girls: emotional adjustment was strongly related to parenting for girls, but it was related to the kind of discipline adopted for boys [56]. Parenting quality is a protective factor for high risk sexual behavior in girls, but for boys, parental overprotection is more efficient [57]. A child’s gender seems to be a moderator for the risk of psychopathology transmission [58].

In a study conducted by Eun and colleagues [35], differences were found in both maternal and paternal care and control and the development of psychopathologies, such as high maternal levels of care and low maternal control being protective for depression, and paternal levels being protective for social phobias and addictions. The same authors also highlighted different associations depending on the gender of the child, e.g., high levels of maternal control associated with the development of anxiety and substance use disorder in daughters, high levels of paternal control correlated with substance use disorder in sons [35].

In our study, the gender influenced the relationship between parental bonding and psychopathology. Only females reported differences in quality of cares: cases girls felt lower care and higher overprotection than controls girls. Boys of the two samples reported similar parental bonding. We suppose a higher vulnerability/sensibility of females to dysfunctional parental bonding, specifically to low maternal care and high paternal overprotection.

In order to investigate the relationship between parental bonding and psychopathology, the sample was divided into four groups according to the presence of internalizing, externalizing, total or no psychopathology, on the basis of self-reported YRS tests.

With regard to paternal bonding, “absent/weak” bonding was the most reported by the individuals with externalizing problems.

More than half of individuals with total problems reported “affectionless control”, while non-pathologic individuals (YSR-score) reported more often “optimal” bonding.

Regarding maternal bonding, the majority of individuals with internalizing problems reported “weak/absent” bonding and about one third “optimal” bonding, both characterized by a low overprotection; the majority of individuals with externalizing problems reported “weak/absent” bonding, that was also the most reported by individual with total problems; non-pathologic individuals reported more often “optimal” bonding.

About the second objective, comparison between the two groups of parents did not reveal any differences: case and control parents reported similar parental bonding with their parents, with the exception of more maternal care experienced by control mothers than by case mothers. Thus, this finding went somewhat against our expectations that control parents would report more optimal relationships, in contrast to case parents, in terms of intergenerational transmission of parental bonding.

Several studies demonstrated the transmission of parental bonding, both as a transmission of parenting characterized by harsh and negative discipline [49,50], as a transmission of parenting characterized by loving and sensitive care [48] and in general both positive and negative parenting [59,60]. These research demonstrated a correlation of moderate degree (0.20 < *ρ* < 0.40) between parenting experienced by parent and parenting subsequently applied to the child. These studies were longitudinal, based on measurements made by experienced clinicians or on the comparison of different types of variables; moreover, parental bonding was assessed during the child’s early childhood, when many external factors (possible moderators) were limited. In our study it was not possible to recognize the intergenerational transmission of the bond and this was probably linked to the use of subjective measures (which therefore referred to perceived experiences) when examining an adolescent population.

However, this result allowed for some reflections on how and why a person does or does not reapply the parenting style they experienced as a child, and what are the mediating and moderating factors of this “legitimate discontinuity” [61]. The main difference we found in the comparison of trans-generational parental ties was that children in control group perceived greater care than their parents; this was not found in cases, with the exception of maternal care which children perceived to be higher than what the mother reported. Thus, in the control group there was a positive change compared to both parents, which was only partially present in the case group. Another difference concerned control (“overprotection”): in the cases, the perceived degree of paternal control increased from one generation to the next. However, when we divided this sample of adolescents into two sexes, this finding was confirmed only in the female subgroup. In the control group, the results differed by parent: children reported more maternal control than their fathers received and less paternal control than their mothers received. When, however, we distinguished the male gender from the female gender, a greater maternal control with respect to the father was a result only among males, while females showed a lesser paternal control with respect to the mother. Thus, there has been a generational shift: mothers controlled their sons more and fathers controlled their daughters less.

A possible interpretation of this result is that a change happened in the generational passage and it differed between the two groups: in the cases, there was not this change or even was negative (greater control perceived by the female children). In the controls, the change occurred and in a positive direction, through increased care, as if the parents would have “learned from experience” by making their child perceived greater affective attention than they themselves had experienced. This represents a proof of the importance of the dimension of care as a protective factor for the development of psychopathology and therefore as a possible therapeutic focus. Moreover, we wanted to highlight how fathers and mothers had different roles: excessive control associated with poor paternal care seemed to be a feature that distinguished subjects with psychopathology from healthy ones; there was less difference between cases and controls about maternal care received, instead.

Furthermore, we considered the matching of parental care in the couple and how it maintained or changed as we moved from one generation to the next. The hypothesis we suggested was that the consistency of the proposed parental pattern would affect the development of the child. We found that in the control group there was always a positive correlation between the degree of care (and control) provided by father and that one provided by mother, so both parents and children had connected parenting patterns. In the case group, this did not occur, in particular about care perceived by children (G2 vs. G1) and control perceived by fathers (G2 vs. G1). Therefore, fathers perceived as hyper-controlling (G2 vs. G1), reported as children (G2 vs. G1) degrees of maternal and paternal control that were not correlated with each other; moreover, subjects presenting psychopathology (G2) reported different degrees of care by father and mother. In the control group, representing the healthy population, on the other hand, the correlation between care provided by father and mother was not only maintained, but strengthened in the transgenerational transition.

Considering the theory of internal operating models and attachment [25], which proposed that parents learn from the parental relationship they have experienced in order to elaborate it and create their own parental characteristics, this appeared to be different in cases and in controls. In families where the child developed psychopathology, it seemed that this process of elaboration did not lead to transmission of characteristics that proved to be protective for the psychopathology (high care and low control), or did not modify parental relationships experienced, even increasing the negative dimensions of the same by increasing control. In the group of controls, on the other hand, a process of “learning from experience” was observed, with reinforcement of the positive aspects (care) and reduction of the negative ones (control), and a correspondence between maternal and paternal care.

This aspect should be explored further, as it reveals that both parents, possibly separately, should be considered when conducting parental bonding studies, and proposing therapeutic goals in clinical settings. An element to be investigated in particular is whether the lack of correlation could be an indicator of conflict or otherwise of educational-relational disconnection within the parental couple.

### Limitations of the Study

The limitations of the study are primarily the small sample size, the use of subjective measures to assess the transmission of parental bonding and also the comparison of children’s questionnaire scores with those of their parents, thus fulfilled at two different developmental stages. However, PBI has been shown to be a highly stable instrument [62].

Another limitation is not having considered parental elements, such as age, couple composition, and the possible presence of psychopathology in order to assess the influence of these variables.

## 5. Conclusions

In our study, we confirmed dated studies concerning the correlation between psychopathology and parental bonding in adolescence, and we also pointed out that it could be more important in the female gender. Individuals with psychopathology experience parental bonding characterized by lower care and a higher overprotection (especially girls), this was not true in the control individuals: we reached the conclusion that these are risk factors, or at least correlated factors, for psychopathology and that can be a target in the therapy of the patients and his family. What we suggest is that in the future research and in clinical environment it would be important the inclusion of both parents, and not only mothers, as often happened. As an example, supporting an environment where the adolescent feels enough care and affection and less overprotection (especially paternal) can be a helpful point in the therapy of the adolescent with psychopathology. Concerning the gender influence, if our findings will be confirmed in further and wider studies, starting a therapy involving parental couple (especially the father) when the patient is a girl, could be an significant clinical indication.

Parental bonding can therefore be considered a protective factor for the development of psychopathology. Therefore, the importance of focusing on parenting also considering transgenerational elements and coherence between parents within the parental bond is highlighted.

## Figures and Tables

**Table 1:** Chi-Squared test PBI bonding.

Parental Bonding	PBI (Paternal Bonding)		Χ^2^ (DoF) *p*-Value	PBI (Maternal Bonding)		Χ^2^ (DoF) *p*-Value
	Controls (%)	Cases (%)	15.174 (3)	Controls (%)	Cases (%)	8.897 (3)
Absent or weak bonding	24.59	30.91	<0.01	34.43	36.36	<0.05
Affectionless control	9.84	34.55		8.20	27.27	
Optimal	47.54	29.09		42.62	27.27	
Affectionate constraint	18.03	5.45		14.75	9.09	

DoF (degree of freedom); *p*-value (significance associated to Chi-square test).

**Table 2:** The *t*-test cases vs. controls.

PBI	Controls	Cases	*t*-Value	Dof	*p*-Value
	M (SD)	M (SD)			
Paternal care	25.787 (4.719)	21.964 (7.521)	3.322	115	0.001 **
Maternal care	28.246 (4.416)	25.143 (6.901)	2.921	115	0.005 **
Paternal overprotection	11.426 (4.075)	12.071 (5.017)	0.766	115	0.449
Maternal overprotection	12.361 (3.951)	13.268 (5.407)	1.042	115	0.304

M (average); SD (standard deviation); DoF (degree of freedom); *p*-value (significance associated to *t* test); ** α ≤ 0.01.

**Table 3:** The *t*-test female/male controls vs. cases.

PBI	Female			Male				
	Controls	Cases	*t*-Value (DoF)	*p*-Value	Controls	Cases	*t*-Value (DoF)	*p*-Value
	M (SD)	M (SD)			M (SD)	M (SD)		
Paternal care	27.414 (4.190)	20.548 (7.357)	4.401 (58)	0.001 **	24.313 (4.687)	23.720 (7.351)	0.371 (55)	0.718
Maternal care	28.931 (4.806)	24.000 (6.942)	3.178 (58)	0.003 **	27.625 (3.927)	26.560 (6.579)	0.759 (55)	0.459
Paternal overprotection	11.897 (3.623)	13.839 (5.125)	1.684 (58)	0.103	11.000 (4.402)	9.880 (3.892)	1.001 (55)	0.329
Maternal overprotection	12.172 (3.957)	14.710 (5.004)	2.169 (58)	0.037 *	12.531 (3.937)	11.480 (5.353)	0.854 (55)	0.405

M (average); S.D. (standard deviation); DoF (degree of freedom); *p*-value (significance associated to *t* test); * α ≤ 0.05; ** α ≤ 0.01.

**Table 4:** Chi-square test based on YSR score.

Parental Bonding	PBI pd				Χ^2^ (DoF) *p*-Value	PBI md				Χ^2^ (DoF) *p*-Value
%	N.C.	I.P.	E.P.	T.P.	21.513 (9) <0.05	N.C.	I.P.	E.P.	T.P.	21.337 (9) <0.05
Absent or weak bonding	21.54	42.11	46.15	23.53		26.15	42.11	46.15	58.83	
Affectionless control	13.85	21.05	15.39	52.94		10.77	15.79	23.09	35.29	
Optimal	47.69	31.58	23.08	23.53		47.69	31.58	15.38	5.88	
Affectionate constraint	16.92	5.26	15.38	0		15.39	10.52	15.38	0	

PBI pd (paternal bonding); PBI md (maternal bonding); N.C. (non-pathologic); I.P. (internalizing problems); E.P. (externalizing problems); T.P. (total problems); DoF (degree of freedom); *p*-value (significance associated to Chi-square test).

**Table 5:** The *t*-test G1 controls vs. cases.

PBI G2	Fathers				Mothers			
	Controls M (SD)	Cases M (SD)	*t*-Test (DoF)	*p*-Value	Controls M (SD)	Cases M (SD)	*t*-Test (DoF)	*p*-Value
Paternal care	19.849 (6.208)	20.647 (5.973)	0.668 (102)	0.510	22.542 (5.741)	20.468 (7.109)	1.760 (119)	0.083
Maternal care	25.241 (4.826)	24.816 (6.143)	0.392 (101)	0.699	25.426 (5.345)	21.557 (7.352)	3.324 (120)	0.001 **
Paternal overprotection	11.057 (3.848)	9.824 (3.846)	1.634 (102)	0.109	13.339 (4.225)	12.581 (3.892)	1.027 (119)	0.310
Maternal overprotection	10.426 (4.404)	9.480 (3.775)	1.165 (101)	0.249	12.230 (3.637)	11.705 (4.236)	0.734 (120)	0.470

M (average); S.D. (standard deviation); DoF (degrees of freedom); *p*-value (significance associated to *t* test); ** α ≤ 0.01.

**Table 6:** The *t*-test G2 vs. G1.

PBI	Controls					Cases				
	G2	G1 Ft	G2 Mt	G2 vs. G1 Ft	G2 vs. G1 Mt	G2	G1 Ft	G2 Mt	G2 vs. G1 Ft	G2 vs. G1 Mt
	M (SD)	M (SD)	M (SD)	*t*-Test (DoF) *p*-Value	*t*-Test (DoF) *p*-Value	M (SD)	M (SD)	M (SD)	*t*-Test (DoF) *p*-Value	*t*-Test (DoF) *p*-Value
Paternal care	25.787 (4.719)	19.849 (6.208)	22.542 (5.741)	5.791 (112) 0.001 **	3.387 (118) 0.001 **	21.964 (7.521)	20.647 (5.073)	20.468 (7.109)	1.051 (105) 0.326	1.111 (116) 0.273
Maternal care	28.246 (4.416)	25.241 (4.826)	25.426 (5.345)	3.487 (113) 0.001 **	3.177 (120) 0.002 **	25.143 (6.901)	24.816 (6.143)	21.557 (7.352)	0.255 (103) 0.801	2.714 (115) 0.008 **
Paternal overprotection	11.426 (4.075)	11.057 (3.848)	13.339 (4.225)	0.495 (112) 0.624	2.525 (118) 0.014 *	12.071 (5.017)	9.824 (3.864)	12.581 (3.892)	2.577 (105) 0.012 *	0.620 (116) 0.541
Maternal overprotection	12.361 (3.951)	10.426 (4.404)	12.230 (3.673)	2.484 (113) 0.015 *	0.189 (120) 0.851	13.268 (5.407)	9.480 (3.775)	11.705 (4.236)	4.105 (103) 0.000 **	1.748 (115) 0.086

M (average), S.D. (standard deviation); DoF (degrees of freedom); Ft (father); Mt (mother); *p*-value (significance associated to *t* test); * α ≤ 0.05; ** α ≤ 0.01.

**Table 7:** Female/males: *t*-test G2 vs. G1.

**Female**	**PBI**	**Controls**					**Cases**				
		**G2**	**G1 Ft**	**G2 Mt**	**G1 vs. G1 Ft**	**G2 vs. G1 Mt**	**G2**	**G1Ft**	**G1 Mt**	**G2 vs. G1 Ft**	**G2 vs. G1 Mt**
		**M (SD)**	**M (SD)**	**M (SD)**	***t*-Value (DoF)** ***p*-Value**	***t*-Value (DoF)** ***p*-Value**	**M (SD)**	**M (SD)**	**M (SD)**	***t*-Value (DoF)** ***p*-Value**	***t*-Value (DoF)** ***p*-Value**
Paternal care		27.414 (4.190)	19.037 (7.121)	21.963 (6.143)	5.411 (54) 0.001 **	3.903 (54) 0.001 **	20.548 (7.357)	21.179 (6.042)	20.444 (7.131)	0.358 (57) 0.727	0.059 (65) 0.954
Maternal care		28.931 (4.806)	25.000 (5.340)	24.759 (6.295)	2.899 (54) 0.006 **	2.799 (54) 0.007 **	24.000 (6.942)	25.346 (5.519)	19.686 (7.566)	0.818 (57) 0.436	2.417 (65) 0.021 *
Paternal overprotection		11.897 (3.623)	11.556 (3.794)	14.222 (4.677)	0.344 (54) 0.737	2.088 (54) 0.045 *	13.839 (5.125)	9.429 (4.255)	13.361 (4.049)	3.574 (57) 0.001 **	0.426 (65) 0.676
Maternal overprotection		12.172 (3.957)	10.481 (4.810)	12.483 (3.255)	1.441(54) 0.163	0.319 (54) 0.750	14.710 (5.004)	9.111 (4.417)	12.571 (3.857)	4.535 (57) 0.001 **	1.974 (65) 0.001 **
**Male**	**PBI**	**Controls**					**Cases**				
		**G2**	**G1 Ft**	**G1 Mt**	**G2 vs. G1 Ft**	**G2 vs. G1 Mt**	**G2**	**G1 Ft**	**G1 Mt**	**G2 vs. G1 Ft**	**G2 vs. G1 Mt**
		**M (SD)**	**M (SD)**	**M (SD)**	***t*-Test (DoF)** ***p*-Value**	***t*-Test (DoF)** ***p*-Value**	**M (SD)**	**M (SD)**	**M (SD)**	***t*-Test (DoF)** ***p*-Value**	***t*-Test (DoF)** ***p*-Value**
Paternal care		24.313 (4.687)	20.692 (4.952)	23.031 (5.330)	2.853 (56) 0.007 **	1.022 (62) 0.319	23.720 (7.351)	20.000 (5.823)	20.500 (7.078)	1.932 (46) 0.065	1.594 (49) 0.125
Maternal care		27.625 (3.927)	25.481 (4.237)	26.031 (4.217)	1.996 (56) 0.053	1.565 (62) 0.129	26.560 (6.579)	24.217 (6.731)	24.077 (6.220)	1.219 (46) 0.239	1.385 (49) 0.181
Paternal overprotection		11.000 (4.402)	10.538 (3.835)	12.594 (3.639)	0.421 (56) 0.681	1.579 (62) 0.125	9.880 (3.892)	10.304 (3.263)	11.500 (3.377)	0.407 (46) 0.692	1.589 (49) 0.126
Maternal overprotection		12.531 (3.937)	10.370 (3.955)	12.000 (4.000)	2.075 (56) 0.044 *	0.535 (62) 0.600	11.480 (5.353)	9.913 (2.781)	10.538 (4.440)	1.256 (46) 0.225	0.685 (49) 0.505

M (average), S.D. (standard deviation); DoF (degree of freedom); Ft (father); Mt (mother); p-value (significance associated to t test); * α ≤ 0.05; ** α ≤ 0.01.

## Data Availability

Restrictions apply to the availability of these data. Data was obtained from a broader project about family interactions, approved by Institutional Ethical Commission, and are available from the Principal Investigation on reasonable request.
